# Nanoengineered phytochemicals overcome blood–brain barrier constraints in neurodegenerative disorders

**DOI:** 10.3389/fneur.2026.1792829

**Published:** 2026-03-23

**Authors:** Alkusha Naim, Azhar Mahmood Farooqui, Badruddeen, Mohammad Irfan Khan, Juber Akhtar, Asad Ahmad, Sumel Ashique, Anas Islam

**Affiliations:** 1 Faculty of Pharmacy, Integral University, Lucknow, Uttar Pradesh, India; 2 Integral Institute of Medical Sciences and Research, Integral University, Lucknow, Uttar Pradesh, India; 3 Department of Pharmaceutical Technology, Bharat Technology, Uluberia, West Bengal, India

**Keywords:** blood–brain barrier, brain targeting, nanocarriers, nano-phytomedicine, neurodegeneration, phytochemicals

## Abstract

Neurodegenerative disorders represent a growing global health burden and remain largely incurable, with current therapies providing only symptomatic relief and limited disease modifications. A major obstacle to effective treatment is the inability of many neuroprotective agents to reach the brain at therapeutically relevant concentrations due to poor bioavailability and the restrictive nature of the blood–brain barrier. Plant-derived phytochemicals possess well-documented antioxidant, anti-inflammatory, anti-apoptotic, and neuromodulatory activities; however, their clinical translation has been hindered by physicochemical instability, rapid metabolism, and insufficient brain exposure. This review critically examines nanoengineered delivery systems as a strategy to overcome these limitations and enable the effective brain targeting of neuroprotective phytochemicals. By integrating mechanistic insights with preclinical and emerging clinical evidence, we compared lipid-based, polymeric, vesicular, and dendritic nanocarriers, highlighting how particle size, surface chemistry, and ligand functionalization govern blood–brain barrier transport and intracerebral distribution. Particular emphasis is placed on rational design principles that consistently enhance brain bioavailability and therapeutic efficacy across models of Alzheimer’s disease, Parkinson’s disease, multiple sclerosis, and related disorders. Beyond efficacy, we analyzed key translational challenges, including nanocarrier-associated neurotoxicity, standardization of herbal activities, and regulatory gaps unique to herbal nanomedicines. Collectively, this synthesis reframes nano-phytomedicine not as an incremental formulation upgrade but as a design-driven strategy capable of unlocking the therapeutic potential of phytochemicals for neurodegenerative disease management.

## 1 Introduction

Neurodegenerative disorders (NDD) constitute a critical global health burden (1). In 2015, dementia affected approximately 46.8 million individuals worldwide, while Parkinson’s disease (PD) accounted for more than 6 million cases, with the prevalence rising steadily due to population aging (2, 3). The five most prevalent neurodegenerative disorders (NDD) Alzheimer’s disease (AD), Parkinson’s disease (PD), Huntington’s disease (HD), amyotrophic lateral sclerosis (ALS), and multiple sclerosis (MS), currently lack curative interventions (4). These disorders follow a chronic progressive course marked by sustained neuronal degeneration, which culminates in significant functional deterioration. Clinical manifestations frequently involve impairments in motor control and higher cognitive functions, including dementia, leading to a pronounced decline in the patient’s quality of life (5). NDD is defined as progressive age-associated deterioration of neural function, primarily driven by neuronal loss. This sustained decline culminates in severe, long-lasting consequences for both overall health and the quality of life (6).

Currently, the prevalence of neurological disorders is rising sharply, while prolonged use of synthetic agents often results in adverse effects. This shift in scientific interest has led to exploration of natural sources as potential alternatives. Several medicinal plants, including *Ginkgo biloba*, *Panax ginseng*, *Valeriana officinalis*, and *Withania somnifera*, have been widely used in traditional medicine because of their notable neuroprotective potential (1, 7). The neuroprotective efficacy of phytochemicals in maintaining brain health during aging is primarily attributed to multiple mechanisms, including AChE and MAO inhibition, antioxidative and anti-inflammatory activities, antithrombotic and anti-apoptotic properties, and the enhancement of neurotrophic signaling pathways (8). Natural products have been extensively used for the treatment of human ailments (9, 10). Owing to their diverse bioactivities and favorable safety profiles, they have gained significant application in pharmaceuticals, nutraceuticals, functional foods, and therapeutic interventions. Nevertheless, their clinical relevance is frequently constrained by challenges such as low solubility in aqueous media, physicochemical instability, and inadequate oral bioavailability (11). During transit through the GIT, these bioactive compounds are exposed to multiple degradation conditions, including mastication, swallowing, gastric acidity, bile salts, enzymatic hydrolysis, and metabolic transformations. These harsh processes markedly diminish their biological activity and therapeutic effectiveness (12). Currently, therapeutic interventions for NDD such as PD and AD primarily offer symptomatic management rather than disease modification (13). In contrast, the treatment outcomes for brain tumors remain even less favorable. A principal obstacle to successful CNS therapy is the highly selective BBB, which restricts the passage of nearly 98% low-MW compounds and effectively excludes most macromolecular agents from accessing the brain parenchyma (14). The BBB architecture comprises cerebral endothelial cells supported by astrocytes and pericytes, with impermeability predominantly regulated by tight and adherens junction complexes linking brain capillary endothelial cells. These specialized intercellular junctions impart extremely low paracellular permeability and exceptionally elevated transendothelial electrical resistance, which is estimated to be approximately 500-to 600-fold higher than that of the peripheral vasculature (15). Consequently, drug delivery limitations significantly hinder clinical progress (16), and enhancing the stability of these bioactive agents within food matrices and during their passage through the GIT remains a critical challenge (12).

The primary challenge in treating CNS disorders is to achieve effective drug delivery to the brain parenchyma. Three primary routes have been explored: transBBB transport, intranasal administration, and intrathecal delivery. Among these, BBB penetration is the most extensively studied and is considered the most promising strategy because of its minimally invasive nature (17). The limited success of current therapies is largely due to the poor bioavailability of pharmacologically active agents in the brain, primarily due to the restrictive nature of the BBB. To partially compensate for this, patients are often given high systemic doses, which may increase brain exposure but also lead to significant off-target toxicity. This highlights the urgent need for advanced therapeutic strategies that can effectively manage neurological disorders. Nanostructured drug delivery systems have attracted considerable attention as potential solutions (18), with nanotechnology offering transformative progress in the development of more efficient and targeted therapeutics (19). Nanomedicine aims to achieve precise diagnosis and therapy with minimal adverse effects, utilizing nanocarrier submicron colloidal systems, typically below 1,000 nm. Their elevated surface-area-to-volume ratio allows for the modulation of drug physicochemical properties and biological activity (20). Furthermore, nanocarrier systems shield therapeutic agents from physicochemical and biological degradation, including enzymatic breakdown, pH variability, and moisture-induced instability, while optimizing PK and biodistribution via passive or ligand-mediated targeting (21). These attributes collectively enhance the therapeutic performance by minimizing off-target exposure and systemic toxicity (22). Additional benefits include increased bioavailability, regulated and sustained drug release, extended systemic residence time, improved cellular internalization, and tissue- or site-selective delivery (23). Notably, lipid-based, polymeric, and inorganic NPs have been widely explored as non-invasive platforms to enable drug passage across the BBB. Polymeric NPs can be fabricated using either biopolymers or synthetic materials; commonly employed natural polymers include chitosan, cellulose, and gelatin, whereas PLA and PLGA are widely utilized synthetic alternatives (24). Owing to their structural adaptability and generally favorable biocompatibility properties, largely dictated by the selected polymer matrix, these nanoscale systems have attracted considerable research interest (25).

Despite extensive preclinical evidence supporting the neuroprotective potential of phytochemicals, their clinical translation remains limited owing to poor bioavailability and restricted blood–brain barrier transport. Although nanotechnology-based delivery systems have been explored to address these challenges, the current literature lacks mechanistic integration and critical comparison of nanoformulation strategies. This review analyzes the advances in nano-phytomedicine for brain disorders, focusing on how nanocarriers and blood–brain barrier-crossing strategies enhance the efficacy of neuroprotective phytochemicals. Through this comprehensive integration, we critically evaluated current strategies, identified emergent design paradigms, and categorized the most pressing translational challenges, offering a refined understanding essential for accelerating the clinical development of brain-targeted nano-phytochemicals.

### 1.1 What this review adds beyond existing literature

Existing reviews typically treat phytochemical neuroprotection and BBB-targeted nanocarriers as separate topics. Reviews that focus on phytochemicals emphasize mechanisms and preclinical efficacy, while nanoparticle reviews cover transport strategies and materials science, and few syntheses explicitly integrate nano-design variables with BBB transport outcomes for phytochemicals (26, 27). This review adds three practical contributions. First, it maps nano-design variables to measurable BBB outcomes, identifying which features consistently track with improved brain exposure across studies. Second, it provides a compact, cross-platform comparison that highlights reproducible design rules and their mechanistic rationale, for example: (i) a hydrodynamic size window near 50–150 nm often balances endothelial uptake and parenchymal diffusion; (ii) near-neutral or lightly anionic surface chemistry and PEGylation extend circulation while reducing acute cationic toxicity; (iii) receptor-targeting (for example transferrin, lactoferrin, Angiopep-2) reproducibly enhances transcytosis via RMT pathways; and (iv) biodegradable polymer backbones (for example PLGA) and lipid matrices give superior safety and manufacturability compared with many non-degradable inorganic platforms. Each design rule is supported below by comparative evidence and representative primary studies (28–31). Third, unlike prior compilations, we flag translationally critical gaps: scarce head-to-head comparative PK data, inconsistent PK endpoints (free vs. total drug), short safety follow-up, and limited chronic-dosing biodistribution studies. We therefore present concrete, prioritized experiments (short- and long-term PK, standardized brain AUC measures, chronic biodistribution and microglial phenotyping) that would most efficiently validate nano-design hypotheses and accelerate clinical translation (32, 33).

## 2 Phytochemicals for brain health: promise and challenges

### 2.1 Major neuroprotective phytochemicals

#### 2.1.1 Curcumin

Curcumin, a hydrophobic polyphenol isolated from the rhizomes of *Curcuma longa*, has attracted considerable interest as a candidate therapeutic agent for NDD including PD, AD, HD, MS, ALS, and prion-associated disorders. This growing focus is attributable to its broad-spectrum biological activities, including anti-inflammatory, antioxidative, immunomodulatory, neuroprotective, antiproliferative, antibacterial, and anticancer effects (34). Nonetheless, its clinical translation remains limited by its low bioavailability and limited confirmatory evidence from human studies. Despite these limitations, curcumin is increasingly regarded as a promising adjunctive intervention in AD owing to its ability to act through multiple neuroprotective pathways (35). Early phytochemical analyses of methanolic turmeric rhizome extracts have identified a wide array of bioactive constituents, including alkaloids, flavonoids, phenolic compounds, saponins, terpenoids, cardiac glycosides, fixed oils, and fatty acids (36). In an experimental investigation, Abdelrhman et al. reported that curcumin administration significantly improved both cognitive and motor functions in mice compared to disease model controls (*p* < 0.01). Treatment was associated with a 35% reduction in oxidative stress and a 40% decrease in brain inflammatory cytokine levels, while histopathological evaluation demonstrated approximately a 30% attenuation of neuronal damage, highlighting curcumin’s neuroprotective effects mediated through suppression of oxidative stress and neuroinflammation (37, 38). Consistent findings were described by Li et al., who demonstrated that curcumin protected cerebral microvascular endothelial (bEnd.3) and neuronal (HT22) cells exposed to OGD/R, an established *in vitro* model of cerebral IRI. These results further support the role of curcumin in regulating oxidative stress responses and cell survival signaling during cerebral IRI (39). Moreover, ElBini-Dhouib et al. evaluated curcumin in an AlCl₃-induced rat model of sporadic AD and observed significant neuroprotective and restorative effects, including enhancement of antioxidant enzyme activity, upregulation of anti-inflammatory cytokines, suppression of hippocampal apoptosis, and concomitant reduction of pro-oxidant markers (40). In addition, by improving the bioavailability of nitric oxide (NO), the curcumin increases endothelial function, nano-curcumin could potentially accumulate more easily in organs and tissues, resulting in increased staining without significant therapeutic effects, This paradoxical situation in clinical use raises concerns about the true efficacy of curcumin nano-formulations (41).

#### 2.1.2 Resveratrol

RSV, a naturally derived polyphenolic molecule, is abundant in grapes, blueberries, raspberries, mulberries, and peanuts. Its protective roles against cardiovascular pathologies, neurodegenerative disorders, stroke, and epilepsy (42). The pronounced antioxidant properties of the compound, notably its capacity to neutralize mitochondrial ROS, are central to its therapeutic efficacy under CNS-related conditions. In PD, RSV exerts neuroprotective effects by inhibiting *α*-syn aggregation and associated cytotoxicity, decreasing total and oligomeric α-syn levels, and mitigating neuroinflammation and oxidative damage. Its clinical potential in major neurodegenerative diseases is currently being assessed in ongoing trials (43). At the mechanistic level, RSV promotes mitophagy through pathways such as SIRT-1 and AMPK/ERK (44). Preclinical studies have further elucidated RSV’s neuroprotective actions of RSV. Ye et al. demonstrated that RSV (1–10 μM) markedly enhanced the survival of primary cortical neurons exposed to OGD/R injury by attenuating apoptosis, maintaining the mitochondrial membrane potential, and lowering oxidative stress (45). In a complementary study, Zhang et al. revealed that RSV confers dose-dependent protection to cortical neurons against Aβ25-35-induced cytotoxicity, improves cell viability, and reduces apoptotic events via activation of the SIRT1/Akt1 signaling axis (46). Collectively, these findings highlight RSV’s neuroprotective potential of RSV via multi-targeted mechanisms, supporting its efficacy as a therapeutic agent for NDDs. In a study carried out by Shamsher E et al., presented in this paper, it is shown that stable formulations of resveratrol have been developed that have increased bioavailability and greater particle size and stability than previously reported resveratrol nanoparticle formulations. Like other resveratrol nanoformulations developed by the researchers, these resveratrol nanoparticles will also extend the half life and increase the bioavailability of resveratrol. The resveratrol nanoformulations have a micellar structure that protects the resveratrol from enzymatic degradation, increasing its ability to be absorbed and delaying its release from the resveratrol nanoformulation over time (47).

#### 2.1.3 Quercetin

Quercetin (QU), a widely distributed dietary flavonoid, is well recognized for its antioxidant and anti-inflammatory properties and contributes to the prevention of various chronic diseases. Notably, QU has demonstrated significant neuroprotective potential, supporting neuronal survival and function (48). *In vitro* investigations have provided compelling evidence of QU’s protective effects of QU on neuronal cells. Taşkıran and Topçu reported that pretreatment of HT-22 hippocampal neurons exposed to 4-AP-induced stress with QU enhanced cell viability, reduced nuclear apoptotic changes, and suppressed oxidative stress by restoring TAS and lowering TOS. Moreover, QU decreased ER stress markers, including ATF-4 and CHOP, indicating its protective role via modulation of the oxidative and ER stress pathways (49). Similarly, Bao et al. demonstrated that QU protects PC-12 cells and hippocampal neurons from H₂O₂-induced oxidative injury by reducing LDH release, ROS accumulation, lipid peroxidation, and MDA levels. Additionally, QU promotes antioxidant defenses by increasing the activity of CAT, SOD, and GSH-Px, while also modulating apoptotic signaling by upregulating Bcl-2 and suppressing Bax, p53, and cleaved caspase-3 expression (50). Further mechanistic insights were provided by Wang et al., who showed that cerebral ischemia/reperfusion and OGDR reduced ERK and Akt phosphorylation while enhancing phosphatase activity, leading to neuronal injury. QU treatment restores ERK/Akt phosphorylation and suppresses phosphatase activity, thereby preventing apoptosis. Since inhibition of ERK or Akt independently triggers cytotoxicity, these findings highlight QU’s neuroprotective action of QU through the maintenance of ERK/Akt signaling (51). Additionally, Heo and Lee reported that QU provides stronger neuroprotection than vitamin C against hydrogen peroxide-induced PC-12 cell degeneration. Treatment with QU improved cell viability and reduced membrane damage, as evidenced by the decreased LDH release and trypan blue staining (52). With the use of quercetin in nanomaterials, scientists have managed to enhance its stability, bioavailability, and ability to target the brain. These nanocarriers, such as liposomes, nanoparticles, and micelles, can penetrate the BBB effectively, maximizing the therapeutic potential of quercetin. Quercetin-loaded nanoparticles greatly increased the penetration of BBB and neuroprotective activity (53).

#### 2.1.4 Withanolides

*Withania somnifera* L. Dunal (Solanaceae), commonly known as ashwagandha or “Indian ginseng,” constitutes a cornerstone of Ayurvedic therapeutics, traditionally valued for its roles as a neurotonic, memory enhancer, and adaptogen, exhibiting anti-aging, antistress, immunomodulatory, and antioxidant properties. Contemporary pharmacological investigations have confirmed its neuroprotective effects. Kumar et al. reported that the aqueous root extract of *W. somnifera* conferred significant concentration-dependent protection to differentiated PC12 cells against H₂O₂- and Aβ(1–42)-induced cytotoxicity. LC–MS profiling has identified withanolide derivatives, notably withaferin A, as the principal bioactive constituents, implicating withanolides as key mediators in counteracting oxidative stress-induced neurotoxicity (54). Supporting these *in vitro* findings, Manjunath and Muralidhara demonstrated *in vivo* that *W. somnifera* administration mitigated ROT-induced neurotoxicity in Drosophila, enhancing survival, locomotor performance, mitochondrial enzyme activity, and dopamine levels while attenuating oxidative stress and cholinergic deficits (55).

In rodent models, *W. somnifera* exhibits broad-spectrum neuroprotective actions. Alzoubi et al. observed that WS root extract (500 mg/kg/day) prevented memory deficits in a rat model of PTSD, preserved spatial learning and memory functions, and reduced hippocampal oxidative stress. UHPLC analysis identified isowithanone (0.23% w/w) as the major marker compound (56). Similarly, Soman et al. demonstrated that both WS root extract and Withanolide A improved spatial memory in epileptic rats by restoring antioxidant enzyme activity (SOD and CAT), reducing lipid peroxidation, and reversing NMDA receptor downregulation and hippocampal neuronal loss, thereby ameliorating glutamatergic dysfunction (57). Consistent with these observations, Vats et al. reported that WS attenuated oxidative stress in a CUS-induced depression model in Wistar rats, as evidenced by decreased cerebellar lipid peroxidation, restored SOD levels (*p* < 0.001), and enhanced sensorimotor coordination (58). These results underscore *W. somnifera*’s anti-stress and neuroprotective effects of *W. somnifera* on cerebellar oxidative stress and motor functions.

#### 2.1.5 Bacosides

Sangeet & Khan highlighted the potential of *B. monnieri*-derived phytochemicals, particularly Bacopaside I, as potent BACE1 inhibitors, exhibiting enhanced binding affinity and unique interaction profiles relative to synthetic inhibitors such as Atabecestat, Lanabecestat, and Verubecestat. Molecular docking and simulation analyses indicate a more precise inhibitory mechanism, underscoring the therapeutic relevance of natural compounds in AD and advocating the integration of traditional medicinal agents into contemporary drug discovery paradigms (59). Complementing these findings, Rastogi et al. demonstrated that prolonged oral administration of bacosides markedly attenuated age-associated chronic neuroinflammation in the cortical regions of middle-aged and elderly Wistar rats. Bacosides reduce elevated levels of pro-inflammatory cytokines, iNOS expression, total nitrite, and lipofuscin accumulation, suggesting robust neuroprotective effects with the potential to mitigate aging-related neurological decline and progression of SDAT (60). Furthermore, Ghosh et al. reported that *B. monnieri* extract and Bacoside-A confer protection against oxidative stress in neurodegenerative conditions through the modulation of the Keap1-Nrf2 signaling pathway. Both *in vitro* and *in vivo* experiments demonstrated decreased ROS generation, preservation of mitochondrial integrity, reduced apoptosis, and protection of hippocampal neurons, highlighting their potential as natural neuroprotective agents (61). Similarly, purified bacoside A from *Bacopa monnieri* strongly inhibited acetylcholinesterase, reduced Aβ42 aggregation by 78%, and exhibited potent antioxidant activity. With no observed toxicity, it demonstrates dual potential for Alzheimer’s treatment (62). Pandareesh et al. found in a study that *Bacopa monnieri* extract (BME) improved memory in scopolamine-induced amnesic rats by enhancing performance in NOR, EPM, and MWM tests. It restores neurotransmitter balance, reduces oxidative stress, and modulates key proteins (AChE, BDNF, M1 receptor, CREB) in the hippocampus, confirming its strong neuroprotective and cognition-enhancing effects (63). In agreement, Pham et al. showed that BME enhances spatial working memory in adolescent mice, with lasting effects even 4 weeks post-treatment. Bacopaside I, a key compound, stimulates neural progenitor cell proliferation via the Akt and ERK1/2 signaling pathways, indicating its crucial role in BME’s cognitive benefits (64) (Table 1). The use of chitosan nanoparticles can enhance the solubility, stability, controlled release, cellular absorption and delivery to CNS. Chitosan nanoparticles are biocompatible, biodegradable, mucoadhesive and possess antioxidant activity, thus providing additional neuroprotective properties for BM phytoconstituents (65).

**Table 1:** Summary of *in vivo* neuroprotective activity of major phytochemicals.

Phytochemical	Source	Disease model	Neuroprotective mechanism	Outcome	References
Curcumin	*Curcuma longa*	STZ-induced AD rat model	Reduced acetylcholinesterase activity, oxidative stress, GFAP levels	Improved memory, reduced neuroinflammation	(145)
	*Curcuma longa*	MPTP-induced PD mouse model	Antioxidant, mitochondrial protection	Preserved dopaminergic neurons, reduced motor deficits	(39)
	*Curcuma longa*	EAE mouse model (MS)	Anti-inflammatory, immunomodulatory	Reduced demyelination and inflammatory cytokines	(37)
	*Curcuma longa*	Transgenic HD mouse model	Antioxidant, anti-amyloid	Improved motor function, reduced striatal degeneration	(34)
Withanolides	*Withania somnifera (Ashwagandha root)*	Transgenic AD mouse model	Antioxidant, anti-amyloid, anti-inflammatory	Improved cognition, reduced Aβ plaques, restored synaptic function	(54)
	*Withania somnifera*	MPTP-induced PD mouse model	Antioxidant, mitochondrial enzyme restoration	Preserved dopaminergic neurons, reduced motor deficits	(55)
Resveratrol	*Grapes, berries, peanuts*	Transgenic AD mouse model	SIRT1 activation, antioxidant, anti-amyloid	Reduced Aβ accumulation, improved memory and synaptic plasticity	(190)
	*Grapes, berries, peanuts*	Cell & animal models (co-delivered with ceftriaxone in PNPs) (PD)	Enhanced brain permeation, ↓*α*-synuclein & p-tau, ↑neuroprotective proteins	Reduced apoptosis & neurodegeneration	(43)
Quercetin	*Fruits & vegetables*	APP/PS1 transgenic AD mice	Antioxidant, anti-inflammatory	Improved cognition, reduced Aβ deposition, suppressed microglial activation	(48)
	*Fruits & vegetables*	6-OHDA induced rat model (PD)	Antioxidant, mitochondrial protection	Preserved dopaminergic neurons, improved motor deficits	(50)
Bacosides	*Bacopa monnieri*	Aged rat dementia model	Antioxidant, Nrf2/Keap1 activation, anti-inflammatory	Enhanced learning & memory, reduced oxidative stress and apoptosis	(63)
	*Bacopa monnieri*	β-amyloid-induced neurotoxicity in PC12 cells (AD)	Antioxidant, mitochondrial protection	Prevented neuronal death, restored mitochondrial membrane potential	(62)
	*Bacopa monnieri*	LPS-induced neuroinflammation in mice	Anti-inflammatory, neurogenesis	Reduced neuroinflammatory cytokines, enhanced hippocampal neurogenesis	(61)

### 2.2 Limitations of conventional formulations

#### 2.2.1 Low oral bioavailability

Plant-derived phytochemicals have historically provided substantial therapeutic value and are used to prevent, alleviate, and treat a wide array of diseases. However, numerous bioactive phytoconstituents exhibit limited aqueous solubility and suboptimal oral bioavailability (66). Additionally, the selective nature of the BBB poses a critical barrier, restricting the delivery of these compounds to the CNS and complicating their clinical translation to neurological disorders. This barrier serves to shield neural tissues from xenobiotics and neurotoxic metabolites, thereby maintaining CNS homeostasis (67). As a result, the BBB is a significant hurdle for effective neurotherapeutic delivery, including that of phytochemicals. Conventional drug formulations often fail to overcome this challenge, limiting the cerebral bioavailability of compounds, such as curcumin (68). Pharmacologically, bioavailability refers to the fraction of an ingested substance that reaches systemic circulation and is subsequently distributed to target tissues to exert biological effects. The therapeutic potential of many nutraceuticals remains underexplored because of their low or inconsistent oral bioavailability (69). The poor aqueous solubility of pharmacoactive molecules restricts their pharmacodynamic and pharmacokinetic profiles, including absorption, protein binding, distribution, and overall efficacy, thereby necessitating strategies to enhance their bioavailability. Molecules with limited solubility are at greater risk of failure during drug development. Given that oral dosage forms constitute over 50% of all pharmaceutical products, achieving sufficient solubility is critical for effective delivery and therapeutic action at the target sites (70). Furthermore, inadequate oral bioavailability often results in variable and poorly controlled plasma drug concentrations, which can compromise the efficacy and increase treatment costs (71). For example, diterpenes are limited by factors such as poor water solubility, slow dissolution, low gastrointestinal absorption, chemical and metabolic instability, and rapid elimination (72). According to the Biopharmaceutics Classification System, Class II and IV APIs are characterized by poor solubility and low bioavailability coupled with reduced dissolution rates (70). Consequently, limited aqueous solubility remains a major challenge for achieving consistent and effective therapeutic responses following oral administration (66).

#### 2.2.2 Poor brain accumulation

The BBB is a major impediment to effective drug delivery in the CNS. Within the brain microvasculature, endothelial cells, astrocytic end-feet, and pericytes form a selective interface between the systemic circulation and the CNS. Tight junctions among endothelial cells establish a physical barrier, whereas efflux transporters contribute to a functional transport barrier by actively regulating molecular passage. Compounds with molecular weights below 400 Da and fewer than eight hydrogen bonds can traverse the BBB. However, many therapeutic agents fail to achieve adequate CNS concentrations because their predicted lipophilicity (log P) or lipophilic permeability coefficient (log D) often exceeds the actual permeability observed for lipophilic compounds in the brain. As a result, the number of ND therapeutics progressing to clinical evaluation remains limited, particularly for small molecules and macromolecules including peptides, antibodies, and siRNAs (73).

#### 2.2.3 Rapid metabolism and clearance

**Figure 1:**
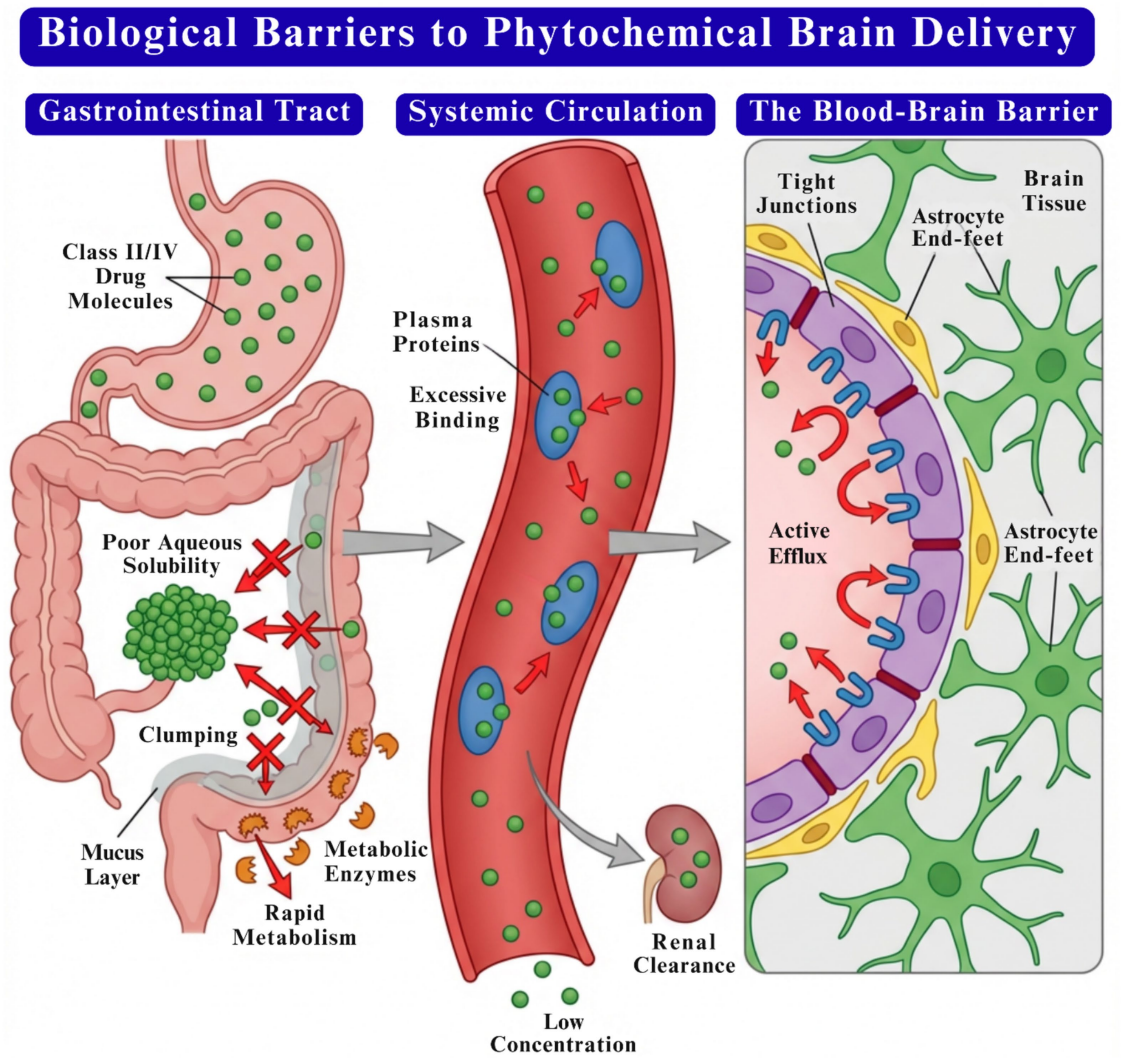
Major biological barriers limiting phytochemical delivery to the brain, including gastrointestinal instability and metabolism, plasma protein binding and rapid systemic clearance, and restricted transport across the blood–brain barrier due to tight junctions and active efflux mechanisms.

Numerous natural products display potent pharmacological effects *in vitro* but often fail to translate these effects *in vivo* due to inadequate gastrointestinal absorption (11). Additional factors limiting the bioavailability of nutraceuticals include poor solubility in gastric fluids, restricted release from the food matrix, limited permeability across epithelial cells or mucus layers, interactions leading to the formation of insoluble complexes with other gastrointestinal constituents, and chemical or enzymatic modifications within the GIT (69) (Figure 1).

## 3 Nanoformulation strategies for phytochemicals

### 3.1 Lipid-based systems

Lipid-based nanocarriers are among the most widely investigated platforms for enhancing the brain delivery of phytochemicals in neurodegenerative disorders, as they offer improved solubility, stability, and pharmacokinetic performance (74).

#### 3.1.1 Solid lipid nanoparticles (SLNs)

Solid lipid nanoparticles (SLNs) are first-generation lipid nanocarriers (30–1,000 nm) composed of a solid lipid matrix (e.g., stearic acid and glyceryl monostearate) stabilized by surfactants such as Tween 80 or Poloxamer, offering biocompatibility, scalability, and controlled release of phytochemicals targeting neurodegenerative disorders (75). Unlike polymeric nanoparticles, SLNs utilize physiological lipids to evade RES uptake, achieve higher drug loading (up to 80% for lipophilics like curcumin), and facilitate BBB penetration via size-dependent endocytosis or paracellular transport, with optimal diameters <200 nm yielding 2-5-fold brain uptake enhancements in rodent models (76, 77). Curcumin-loaded SLNs exemplify this potential, demonstrating superior neuroprotective efficacy in MPTP-PD mice by preserving dopaminergic neurons, restoring SOD/CAT (↑35–50%), reducing motor deficits via sustained release (90% over 24 h) and 3-fold brain AUC increase compared to free curcumin (78). Similarly, naringenin-SLNs (∼20 nm) suppressed autophagy markers and enhanced mitochondrial function in STZ-PC12 cells, outperforming free drugs by mitigating ROS and apoptosis through PINK1/Parkin pathway modulation (79). Capsaicin extract SLNs (200 nm, 80% EE) attenuate H₂O₂-induced ROS in SH-SY5Y cells, confirming PD-relevant neuroprotection with Korsmeyer-Peppas release kinetics (80). *α*-Asarone-SLNs further validated brain targeting, achieving significantly elevated plasma/brain levels post-IV administration versus free drug, ideal for AD/PD therapeutics. T ese systems protect labile phytochemicals from GI degradation/enzymatic hydrolysis, with stearic acid matrices preventing polymorphic transitions for a 30-day stability at 4 °C. Surface modifications (e.g., chitosan coating) enhance mucoadhesion and ligand conjugation (e.g., lactoferrin for TfR-mediated transcytosis), boosting CNS delivery by 4–6 fold in Zebrafish AD models (81). Despite their advantages, SLNs face gelation risks during storage and the burst release of high-melting lipids. Imperfect lattices limit hydrophilic loading to <5%. Preclinical dominance persists, yet Phase I trials (e.g., curcumin-SLNs for MCI) report safe PK with modest cognitive gains, underscoring translational promise amid 2025 nano-antioxidant scoping reviews calling for standardized lipid sourcing and GMP scaling (82). Overall, SLNs position phytochemicals as viable NDD candidates, bridging the Ayurvedic heritage with precision nanomedicine.

#### 3.1.2 Nanostructured lipid carriers (NLCs)

Nanostructured lipid carriers (NLCs), second-generation lipid nanoparticles (10–500 nm), overcome SLN limitations by blending solid lipids (e.g., Compritol 888 ATO) with liquid lipids (e.g., oleic acid, 10–30% ratio), creating imperfect crystal lattices for superior drug loading (up to 81% EE), payload stability, and biphasic release profiles that are ideal for phytochemical brain delivery. This matrix disrupts lipid ordering and prevents expulsion of lipophilic actives, such as curcumin or resveratrol, during storage, while enabling higher hydrophilic encapsulation and scalability via hot homogenization or nano-template engineering (83, 84). These advanced delivery systems offer greater stability than the conventional SLNs. Nanoscale drug design has several advantages including enhanced immunogenicity, improved bioavailability, potential for alternative administration routes, reduced adverse effects, optimized biodistribution, and prolonged drug half-life (19). Sakamula et al. investigated the neuroprotective potential of αM and its nanostructured lipid carrier formulation (αM-NLCs) in a PD mouse model. The αM-NLC formulation conferred superior neuroprotection, preserving neuronal populations in both the striatum and motor cortex, indicating that lipid carriers potentiate the efficacy of αM against oxidative stress and neurodegeneration (85). Similarly, Halder et al. developed and optimized MY-loaded NLCs (MY-NLCs) in an AD rat model, achieving a particle size of approximately 90 nm, a high entrapment efficiency (~81%), and biphasic drug release. *In vitro* studies confirmed efficient cellular uptake, while *in vivo* evaluations revealed 2.77-fold higher plasma exposure and enhanced cerebral targeting compared to free MY (86). Khan et al. assessed the cytotoxicity and cell viability effects of RV and KI, alone and in combination, on Neuro-2a cells. Their results demonstrated that dual drug-loaded NLCs improved the delivery efficiency and therapeutic outcomes, highlighting their potential for AD management (87). The challenges that persist are potential lipid oxidation (mitigated by tocopherols) and burst release in imperfect matrices. Preclinical trials dominate, but 2025 intranasal NLC pilots have reported safe PK with cognitive gains in MCI/PD.

### 3.2 Polymeric nanoparticles

#### 3.2.1 PLGA nanoparticles for sustained drug release

PLGA, a commercially available synthetic copolymer derived from lactic and glycolic acids, has received regulatory approval from the FDA and EMA. PLGA-based NPs represent a promising platform for next-generation therapeutics. Owing to their biocompatibility and biodegradability, they provide drug protection, enhance systemic bioavailability, and enable active targeting for precise, site-specific delivery. Such attributes have been leveraged for the administration of bioactive compounds, including thymoquinone and curcumin, directly to the brain, while preserving their neuroprotective functions. Additionally, these nanoparticles have demonstrated therapeutic actions such as normalization of lysosomal pH in PD models (88).

Wang et al. developed biodegradable PEG-PTMC polymeric nanoparticles (PPNPs) for the delivery of GB in PD therapy. GB-PPNPs achieved sustained release over 48 h, protected neurons from toxic insults, and attained higher plasma and cerebral concentrations than free GB. Treatment with GB-PPNPs improved motor function, dopamine levels, and tyrosine hydroxylase activity in a PD animal model of PD, highlighting the potential of polymeric nanoparticles to enhance CNS drug delivery (89). De Soricellis et al. formulated curcumin-loaded PLGA NPs as a model system for poorly soluble BCS class IV drugs. *In vitro* analysis revealed that lower drug-loaded NPs released 90% of curcumin within 6 h, whereas higher-loaded NPs exhibited sustained release over 7 days, influenced by polymer degradation kinetics and drug–polymer surface interactions, illustrating the scalability and adaptability of the method for tailored drug delivery applications (90). Similarly, Yekeler et al. incorporated curcumin-loaded PLGA NPs (CNPs) into 3D-printed sodium alginate/gelatin scaffolds (CNPSGS) for sublingual administration in AD therapy. The CNPs exhibited controlled curcumin release (99.6% over 18 days) and maintained a high cell viability (>84%) without cytotoxic effects. PCR analyses confirmed that the neuroprotective activity was mediated through modulation of the Wnt/*β*-catenin and PI3K/Akt/GSK-3β signaling pathways (91). Challenges include an acidic microclimate inducing protein aggregation (neutralization with MgCO3), incomplete release of hydrophilics, and scale-up variability. Preclinical superiority approaches such as bacoside-PLGA reverse scopolamine amnesia, but Phase I/II trials (e.g., NanoCur for MCI) show modest efficacy, urging GMP optimization and combination therapies amid 2025 nanomedicine pushes (92–94). PLGA bridges phytochemical instability with chronic CNS dosing, advancing Ayurvedic activities to the clinic.

#### 3.2.2 Chitosan nanoparticles for mucoadhesive properties

Chitosan, a naturally occurring linear amino polysaccharide composed of glucosamine and N-acetylglucosamine residues, possesses free amine groups that facilitate nanoparticle formation via crosslinking or spontaneous self-assembly (95). Among the various NP-based delivery platforms, chitosan-based NPs (CS-NPs) have demonstrated exceptional efficacy in enhancing the oral bioavailability and pharmacological activity of diverse phytochemicals. Encapsulation within CS-NPs confers multiple advantages, including improved aqueous solubility, protection from enzymatic degradation and pH variations in the GI tract, controlled and sustained release of phytoconstituents, and enhanced intestinal absorption through prolonged GI residence mediated by mucoadhesive properties, collectively augmenting the oral bioavailability and bioactivity (96). The presence of hydroxyl (-OH) and amino (-NH₂) groups in chitosan enables hydrogen and covalent bonding, which is critical for its mucoadhesive capabilities (97). CS-NPs, therefore, emerge as biodegradable yet robust carriers for CNS-targeted therapeutics, with all degradation products being non-toxic, non-immunogenic, and non-carcinogenic (95). Moreover, chitosan-based nanostructures can enhance multiple aspects of drug performance, including cellular transport and other biological activities (98).

For instance, Mahanta et al. developed mannose-conjugated chitosan-coated PLGA nanoparticles (CHTMAN-PLGA) for the targeted cerebral delivery of CBD and BDNF. These nanoparticles, designed to engage GLUT-1 receptors on the BBB, exhibited an average particle size of 306 nm, a high positive zeta potential, and sustained CBD release over 22 days. The system efficiently encapsulated plasmid DNA, resulting in a four-fold increase in BDNF expression across various brain cell lines, and demonstrated favorable cytocompatibility and hemocompatibility (99). In a complementary approach, Jiang et al. engineered targeted nanoparticles using carboxymethyl chitosan (CTS) and Jiuzao glutelin isolate (JGI) conjugates to encapsulate RES and QUE. These nanoparticles significantly improved cellular internalization via folic acid receptor-mediated endocytosis and effectively mitigated LPS- and DSS-induced inflammation by modulating the NF-κB/IkBa/AP-1 and AMPK/SIRT1 signaling pathways. These results highlight the potential of these nanoparticles as oral delivery systems for unstable bioactive compounds (100).

### 3.3 Liposomes and phytosomes

Liposomes and phytosomes (30–150 nm) are phospholipid-based vesicular carriers that enhance phytochemical solubility, stability, and BBB permeation for neurodegenerative therapy, with liposomes offering aqueous/lipid bilayer entrapment and phytosomes providing stoichiometric complexation via phospholipid-polyphenol H-bonds.

Liposomes, ranging from 50 to 200 nm in size and composed of phospholipids similar to cellular membranes, are widely regarded as one of the most effective nanocarriers for drug delivery. Their inherent biocompatibility and structural stability allow the encapsulation of both hydrophilic and lipophilic compounds, providing protection against enzymatic and chemical degradation. Liposomes are extensively employed in pharmaceutical applications owing to their high drug-loading capacity and delivery efficiency. They enable controlled release, site-specific targeting, enhanced therapeutic outcomes, and a reduced dosing frequency (101). Encapsulation within the lipid bilayer improves solubility and facilitates targeted delivery to the affected tissues (102). Chen et al. developed glucose-modified quercetin liposomes (QU-Glu-Lip) to address the poor aqueous solubility, limited bioavailability, and restricted brain penetration of quercetin. These liposomes exhibited a high encapsulation efficiency, uniform particle size, and robust stability. Glucose conjugation facilitates GLUT1-mediated transport across the BBB, resulting in increased neuronal uptake and pronounced neuroprotective effects (103). Similarly, Mihoub et al. reported that curcumin encapsulated in RGD peptide-PEGylated nanoliposomes derived from salmon lecithin exhibits improved stability, neuronal compatibility, and neuroprotective efficacy. The nanoliposomes were spherical, nanosized, and negatively charged and effectively mitigated Aβ-induced cytotoxicity in neuronal cells compared to free curcumin (104).

Phytosomes, which are derived from the combination of plant extracts or hydrophilic phytoconstituents with phospholipids, represent a patented lipid-compatible delivery system. The term “phyto” denotes plant, while “some” refers to a cell, reflecting the molecular complexation of plant compounds with lipids. These formulations enhance absorption and bioavailability and offer superior pharmacokinetic and pharmacological profiles relative to conventional preparations (105). These formulations exhibited improved pharmacological and pharmacokinetic properties compared to the prevalent preparations. Phytosomes have remarkable benefits such as high drug encapsulation, a better stability profile, and better bioavailability. Moreover, a higher absorption rate leads to a lower dosage of active constituents to exert a biological effect, as well as polar phytoconstituents (105). It is an innovative formulation for herbal extracts that demonstrates superior absorption compared to conventional herbal extracts. Phytosomes may be effectively absorbed through both the skin and gastrointestinal system, thereby improving bioavailability, which leads to a decreased frequency of dosing compared to conventional herbal extracts (106). Pawar and Bhangale developed *curcumin*-loaded nano-phytosomes using phospholipid Phospholipon 90 H to enhance *curcumin*’s stability and bioavailability of curcumin. Phytosomes prepared at a 1:2 *curcumin*-to-lipid ratio were spherical with particle sizes under 100 nm and exhibited good entrapment efficiency. This formulation demonstrated sustained *curcumin* release, suggesting improved absorption, reduced elimination, and enhanced antioxidant potential (107). Both systems protect against GI/enzymatic degradation, liposomes enable co-encapsulation, and phytosomes simplify scale-up. Challenges include liposome instability (oxidation; tocopherol mitigation), phytosome standardization (HPLC markers essential), and RES uptake by PEG (78).

### 3.4 Nanoemulsions and micelles

#### 3.4.1 Nanoemulsions improve solubility and absorption

Emulsion-based approaches are widely employed to reduce the particle sizes of both hydrophilic and hydrophobic bioactive molecules. Nanoemulsions facilitate improved oral delivery by enhancing solubility and bioavailability, thereby enabling a more efficient traversal of physiological barriers. Notably, NEs function as multifunctional lipid-based nanosystems that combine enhanced permeability, tissue and cellular targeting, imaging, and therapeutic efficacy. The application of NE technology enables the formulation of PHY-based systems with particle sizes typically ranging from 100 to 500 nm, exhibiting superior solubility, physicochemical stability, bioavailability, and prolonged systemic half-life (108). Owing to these advantages, NEs have been extensively explored for drug delivery in neurological disorders including AD, PD, and prion diseases. Their ultrasmall droplet size supports Brownian motion, prevents droplet aggregation, and ensures long-term dispersion stability. Additionally, the large interfacial surface area of NEs facilitates the efficient delivery of active agents across biological barriers, including transdermal routes (109).

Abomosallam et al. investigated the neurotoxic effects of penconazole (PEN) and assessed the neuroprotective efficacy of WSLE formulated as a NE in rats. PEN exposure results in behavioral deficits, elevated oxidative stress, neuroinflammation, neuronal degeneration, and apoptosis in the brain tissue. LC–MS/MS profiling identified 91 bioactive constituents in WSLE. Administration of WSLE NE markedly improves behavioral outcomes and histopathological features compared to WSLE alone by enhancing antioxidant enzyme activity, suppressing pro-inflammatory cytokines, and downregulating neurotoxicity-associated gene expression (110). Similarly, Júnior et al. compared the neuroprotective efficacy of curcumin-loaded nanoemulsions (NC) with free CUR in a rotenone-induced PD mouse model. Both formulations ameliorated motor dysfunction, reduced oxidative damage, and preserved mitochondrial complex I activity in the brain tissue; however, the nanoemulsion formulation demonstrated superior efficacy in preventing motor impairments and mitochondrial dysfunction relative to free CUR (111). Furthermore, Jindal and Singh employed computational approaches alongside oil-in-water nanoemulsion systems to enhance brain delivery of *B. monnieri* and *W. somnifera* extracts. These nanoemulsions exhibit significantly greater neuroprotective effects than the corresponding free extracts, indicating their potential as effective therapeutic strategies for AD management (112).

#### 3.4.2 Micelles for enhancing oral delivery of polyphenols

Polymeric micelles are self-assembled nanostructures formed from amphiphilic polymers in aqueous environments that offer several advantages over conventional DDSs. Their amphiphilic architecture enables the sequestration of poorly water-soluble compounds within a hydrophobic core, while maintaining aqueous stability. Owing to their nanoscale dimensions (10–100 nm), micelles exhibit enhanced penetration through the permeable vasculature via the EPR effect, enhancing their utility in targeted delivery applications, particularly in pathological tissues (113).

### 3.5 Dendrimers and hybrid nanocarriers

**Figure 2:**
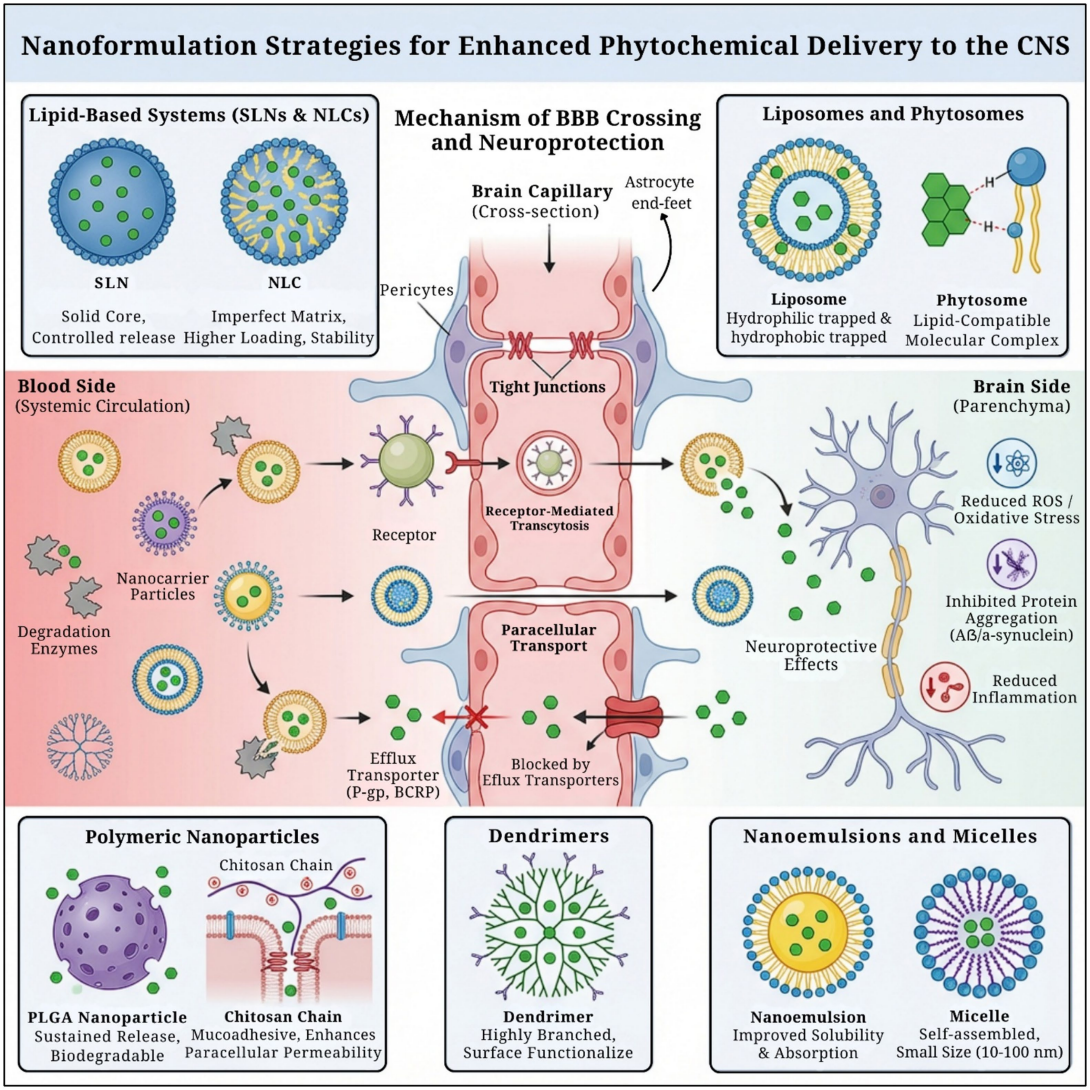
Overview of nanoformulation strategies enabling enhanced phytochemical delivery to the central nervous system through protection from systemic degradation, modulation of efflux transporters, and improved blood–brain barrier crossing, resulting in neuroprotective effects within brain parenchyma.

Dendrimers are highly branched globular macromolecules characterized by a densely functionalized surface and a well-defined internal architecture. Their uniform molecular weight (MW), monodispersity, tunable size, and high density of terminal functional groups render them highly suitable as advanced drug-delivery platforms. The distinct chemical microenvironment between the dendrimer core and periphery enables efficient encapsulation of hydrophobic agents with suboptimal pharmacokinetic and pharmacodynamic profiles (114). Dendrimers can entrap therapeutic molecules through non-covalent interactions and function as solubilizing agents or stabilizers while providing protected compartments for systemic transport, thereby enhancing drug bioavailability (115). In addition, covalent conjugation of bioactive compounds to surface functional groups allows precise control of drug loading, release kinetics, and targeted delivery (114). A wide spectrum of dendrimer architectures have been developed through selective surface functionalization using groups such as –OH, –SH, –NH₂, –COOH, azido, and allyl moieties, as well as through conjugation with carbohydrates, peptides, ligands, or other targeting motifs. Surface modification facilitates the exploitation of endogenous transport mechanisms, thereby enabling dendrimer-mediated traversal of the BBB and enhancing the CNS delivery efficiency (115). Moreira et al. synthesized generation 3 dendrimers and evaluated their effects on amyloid beta (Aβ1-42) fibrillation, a key factor in Alzheimer’s disease. The dendrimers modulated Aβ aggregation in a concentration-dependent manner; at low ratios, they inhibited fibril formation, whereas at higher ratios, they promoted fibrillation. Importantly, they are nontoxic to neuroblastoma cells. These preclinical evidence suggest that dendrimers are promising agents that interfere with Aβ fibrillation, warranting further research on their mechanisms (116). Biophysical characterization and *in vitro* assays demonstrated that DG4.5 enhances curcumin solubility and stability through encapsulation within dendrimer cavities and interactions with terminal carboxyl groups. The resulting CUR-DG4.5 complexes exhibited good biocompatibility, improved cellular uptake, antioxidant activity evidenced by DPPH radical scavenging, protection against H₂O₂-induced oxidative stress, and effective interference with *α*-synuclein aggregation (117) (Figure 2). Table 2 aims to yield a direct comparison between various nanoformulations and address BBB transport mechanism, toxicity profile along with translational considerations.

**Table 2:** Comparative evaluation of nanoformulations for BBB delivery and translational considerations.

Nano formulation	Drug loading capacity and stability	BBB transport mechanism	Toxicity profile	Translational readiness	References
SLNs	Moderate & High	Passive diffusion, adsorptive-mediated	Low	Emerging	(77)
NLCs	High	Enhanced lipid-mediated transport	Low–Moderate	Emerging	(19)
PLGA NPs	Moderate & Very High	Endocytosis, receptor-mediated (if targeted)	Very Low	Advanced	(90)
Chitosan NPs	Moderate	Mucoadhesion, tight junction modulation	Low	Emerging	(100)
Liposomes	Moderate	Fusion, receptor-mediated, adsorptive-mediated	Low	Most advanced	(102)
Phytosomes	Moderate	Lipid phytoconstituent complexation	Low	Early-stage	(105)
Nanoemulsions	Moderate	Lipid-mediated passive transport	Low	Emerging	(109)
Dendrimers	High	Receptor-mediated, adsorptive-mediated	Dose-dependent toxicity	Limited (toxicity concerns)	(114)

## 4 Strategies to cross the blood–brain barrier (BBB)

### 4.1 Physicochemical approaches

Optimizing nanoparticle size and surface charge is a fundamental physicochemical strategy to enhance brain delivery by exploiting the BBB’s tight junction architecture and endothelial transport pathways. Particles smaller than 200 nm more readily undergo adsorptive or receptor-mediated transcytosis and show reduced recognition by the reticuloendothelial system, whereas larger carriers (>200–250 nm) are prone to rapid clearance and poor microvascular penetration in brain capillaries (118, 119). For most nano-phytomedicines, an optimal hydrodynamic diameter of 50–150 nm balances circulation time, endothelial uptake, and diffusion within the brain parenchyma, which is why SLNs, NLCs, PLGA NPs, and liposomes designed for CNS targeting typically fall within this range (118).

Surface charge profoundly influences both BBB interactions and toxicity. Slightly positive or near-neutral zeta potentials (approximately −10 to +10 mV) are generally preferred for systemic administration because highly cationic particles can strongly bind to the negatively charged endothelial glycocalyx and plasma proteins, causing aggregation, complement activation, and membrane damage (120). Nevertheless, a controlled positive charge is advantageous for intranasal and nose-to-brain delivery. Chitosan- or PEI-coated nanoformulations leverage protonated amines to interact electrostatically with sialic acid-rich mucins, improving mucosal adhesion, residence time, and paracellular opening, thereby enhancing transport along the olfactory and trigeminal pathways (121, 122).

For example, resveratrol-loaded solid lipid nanoparticles (SLNs) were characterized as having a homogeneous size distribution and mean diameter of approximately 155 nm and no significant size differences due to resveratrol incorporation. All SLNs had average sizes below 200 nm, making them suitable for prolonged circulation and increased interaction with the BBB, which also allowed them to evade filtration by the liver and spleen. These attributes significantly augment their suitability for brain-specific drug delivery applications (123). In this context, Bana et al. reported that liposomes dual-functionalized with phosphatidic acid and a modified ApoE-derived peptide (mApoE-PA-LIP) achieved efficient targeting of Aβ in the brain, highlighting their therapeutic potential in AD management. These liposomes strongly bound to Aβ, inhibited its aggregation by 70%, and disaggregated preformed aggregates by 60%. Importantly, bi-functionalization significantly enhances BBB permeability *in vitro* and *in vivo* by up to five-fold compared to mono-functionalized liposomes because of the optimized size (< 200 nm) and tailored surface properties. These findings highlight physicochemical strategies, such as nanoparticle size (< 200 nm) and surface charge modification, to improve BBB crossing and targeted brain delivery (124). Furthermore, Y.-P. Chen et al. engineered MSNs with precisely controlled particle sizes and surface charges to facilitate BBB translocation without the need for external stimuli or receptor-mediated functionalization. Among the formulations evaluated, MSNs with a diameter of 50 nm and pronounced negative surface charge (~ − 40 mV) effectively traversed the BBB in zebrafish models, whereas larger particles (200 nm) or positively charged MSNs failed to do so. Upon doxorubicin loading, the 50 nm MSNs demonstrated pH-sensitive drug release, resulting in reduced systemic toxicity and enhanced survival in zebrafish. Proteomic analysis of the protein corona indicated that BBB penetration was likely mediated by interactions with transporter-associated proteins including afamin, apolipoprotein E, and basigin (120). A transcellular model to understand how nanoparticle (NP) size (20–100 nm) and charge affect their permeability through the *in vitro* blood–brain barrier (BBB) was developed in a study. The model revealed that the negatively charged surface of the BBB strongly influences NP transport, with positively charged NPs showing enhanced permeability owing to electrostatic attraction, negatively charged MSNs exhibited no detectable cytotoxicity in astrocytes or glioma cell lines (125). These findings highlight the importance of nanoparticle size and surface charge for BBB penetration and sustained brain drug delivery, minimizing peripheral side effects.

### 4.2 Surface functionalization

PEGylation, which involves covalent grafting of PEG chains onto nanoparticle surfaces, represents a widely employed stealth modification strategy that extends systemic circulation time and enhances brain delivery by minimizing opsonization and subsequent clearance by the mononuclear phagocyte system. The hydrophilic, flexible PEG corona forms a steric barrier that limits protein adsorption (called protein corona formation), aggregation, and complement activation, thereby decreasing clearance by the liver and spleen macrophages and increasing the likelihood of nanocarriers reaching the BBB intact. For lipid-based systems such as liposomes, SLNs, and NLCs, PEG (commonly 2–5 kDa, 2–10 mol%) was incorporated as PEG-lipid conjugates, which significantly extended circulation half-life and converted rapid first-pass clearance into more sustained, near-zero-order pharmacokinetics (126, 127). Jenkins et al. investigated how PEGylated nanoparticles interact with major brain cell types, including immune cells (microglia). It demonstrated for the first time that PEGylation helps nanoparticles evade uptake by both immune and non-immune brain cells, enhancing their extracellular bioavailability. These findings also highlight how changes in the protein corona and surface chemistry influence these interactions, providing valuable insights for designing nanoparticle-based therapies targeting neurological diseases (128). In a related investigation, Q. Du et al. systematically evaluated the influence of PEG chain length (2, 3.4, 5, and 10 kDa) on BBB penetration and cerebral targeting using Angiopep-2-functionalized liposomes. Their findings revealed that liposomes bearing shorter PEG chains exhibited enhanced cellular internalization through endocytic pathways, but demonstrated limited BBB translocation due to inefficient exocytosis. Conversely, liposomes modified with longer PEG chains showed markedly improved BBB transcytosis *in vitro* and significantly increased brain accumulation *in vivo* in both healthy and glioblastoma-bearing murine models (129).

The BBB, formed primarily by BECs, exhibits strict impermeability to biologics and macromolecular therapeutics (130). Consequently, essential peripheral substances required for normal brain function must enter the CNS via specialized active transport mechanisms. One such pathway is RMT, wherein ligands selectively bind to receptors expressed on the luminal surface of BBB endothelial cells, triggering internalization of the receptor–ligand complex into vesicular compartments. These vesicles undergo intracellular trafficking and subsequently fuse with the abluminal membrane to release their cargo into the brain parenchyma. Exploitation of RMT pathways has emerged as a clinically validated and promising strategy to enhance BBB permeability and facilitate targeted delivery of biological therapeutics (30). Ligand conjugation exploits endogenous BBB receptors to actively ferry nanocarriers into the brain, with transferrin, lactoferrin, and glucose among the most widely used ligands for receptor-mediated transport. These ligands are typically grafted onto the PEGylated or surfactant-stabilized surfaces of SLNs, NLCs, PLGA nanoparticles, or liposomes via covalent coupling, preserving receptor affinity while maintaining nano-scale size and colloidal stability (130, 131).

For instance, Ruan et al. introduced an innovative nanoparticle platform (DD-MCT) comprising doxorubicin-loaded dendrigraft poly-L-lysine functionalized with mannopyranoside (MAN) and enveloped by acid-sensitive transferrin (Tf) coating. Following the interaction with Tf receptors, the acidic microenvironment induces Tf cleavage, leading to the release of MAN-decorated nanoparticles (DD-M). These nanoparticles subsequently evade endo/lysosomal entrapment and traverse the BBB via GLUT-mediated exocytosis (132). Using a comparable approach, Singh et al. engineered lactoferrin (Lf)-functionalized SLNs encapsulating docetaxel (DTX) to enhance cerebral delivery. Both *in vitro* and *in vivo* evaluations demonstrated that Lf-SLNs exhibited superior cytotoxicity, increased cellular uptake, and markedly elevated brain concentrations of DTX compared to non-modified SLNs and conventional formulations (133). Additionally, curcumin-loaded lactoferrin nanoparticles (CUR-LF NPs) were developed for intranasal brain delivery, achieving a nanoscale particle size (~85 nm) and positive surface charge. This formulation substantially enhanced the nasal absorption of curcumin, exhibiting a 50-fold increase in permeability and providing pronounced neuroprotection against Aβ-induced toxicity in vitro. *In vivo* studies have revealed high brain-targeting efficiency (248.1%) and significant direct nose-to-brain transport (59.7%) following intranasal administration, underscoring the therapeutic potential of this strategy for neuroprotection (134).

Among the various ligands investigated for facilitating BBB translocation, glucose is particularly attractive because of its role as the primary cerebral energy substrate and the high expression of GLUT1 in BCECs relative to other transporters and receptors (135). In this context, Arora et al. developed liposomal nanoparticles surface-functionalized with mannose, a GLUT1 ligand, in combination with cell-penetrating peptides, to enhance targeted BDNF gene delivery for AD therapy. Dual-functionalized liposomes significantly increased BDNF transfection efficiency and elevated synaptic marker expression, including synaptophysin, in neuronal cells under both *in vitro* and *in vivo* conditions (136).

### 4.3 Stimuli-responsive nanocarriers

As delivery platforms, ideal NPs are required to exhibit a high drug-loading capacity, ensure precise transport of therapeutic agents to diseased tissues and/or target cells without premature leakage, and enable rapid and enhanced drug release at the site of action. To achieve these objectives, a wide range of “smart” polymeric NPs have been developed that respond to specific internal or external stimuli, including variations in the pH, redox potential, temperature, magnetic fields, and light. Such stimulus-responsive nanocarriers have demonstrated enhanced drug release characteristics, to varying extents, in both *in vitro* and *in vivo* settings (137).

pH-responsive nanoparticles exploit the acidic microenvironment of endosomes/lysosomes (pH 5.0–6.5) versus physiological pH (7.4), enabling endosomal escape or triggered payload release post-BBB transcytosis. Polymeric systems such as PDEAEMA (protonates below pH 6.5, swelling/disintegration) or PDMAEMA nanogels loaded with curcumin undergo rapid size expansion (>2x) and 46% release at pH 5.5, facilitating cytosolic delivery, while being stable at pH 7.4. Dual pH/light-responsive PEG-PDMAEMA-PNBAE micelles released curcumin at 46.5% under UV light at pH 5.5, protecting PC12 cells from Aβ-induced apoptosis (138). pH-sensitive TfR bispecific antibodies (e.g., pH-PEG engager-TfR) dissociate PEG-NPs in acidic endosomes, boosting liposomal doxorubicin brain accumulation 2-3x in GBM xenografts (139).

Among these, redox-responsive systems are particularly appealing because of the pronounced redox gradients that exist in biological environments. Systemic circulation is generally characterized by an oxidizing milieu, whereas intracellular compartments exhibit substantially more reducing conditions, creating opportunities for selective and site-specific cargo release. Importantly, experimental findings indicate that redox regulation is not uniform; rather, distinct cellular compartments display subtle variations in redox potential and the identity and concentration of reducing agents differ across these microenvironments. These complexities underscore the need for tailored redox-sensitive nanocarrier design to achieve optimal therapeutic precision (140). Redox-responsive systems leverage elevated glutathione (GSH, 1–10 mM intracellular vs. 2–20 μM extracellular) levels to cleave disulfide bonds and disintegrate carriers for burst release. Cystamine-crosslinked PDEAEM-PEG nanogels degrade >80% in 0.5 h with 10 mM GSH, releasing curcumin for ROS mitigation in neuronal cells. CSO-ss-CUR (chitosan-curcumin disulfide) self-assembles into NPs that cross the BBB and rapidly destabilize intracellularly, thus enhancing AD neuroprotection (141).

**Figure 3:**
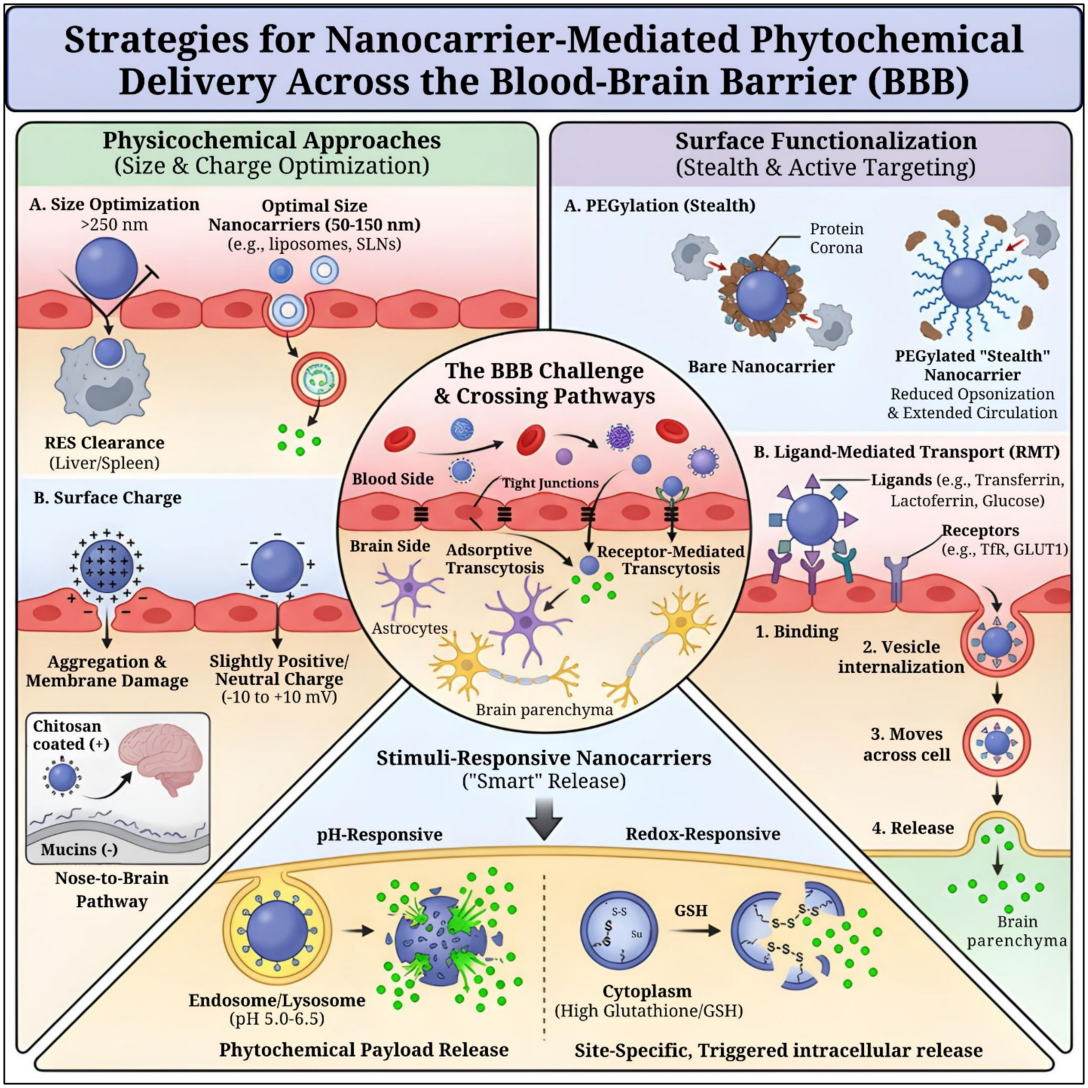
Schematic representation of physicochemical optimization, surface functionalization, and stimuli-responsive strategies that enable nanocarrier-mediated transport of phytochemicals across the blood–brain barrier, facilitating targeted brain delivery and site-specific intracellular release.

Redox-sensitive micelles co-deliver TMZ/*β*-lapachone to GBM, cleaving disulfides in GSH-rich tumors, whereas pH triggers endosomal escape (142). This ensures that phytochemicals, such as curcumin, remain intact during circulation/BBB transit but unleash at disease sites (acidic/inflamed brain regions), amplifying Nrf2/antioxidant effects in AD/PD models with minimal off-target toxicity. Preclinical data highlight 3-5x efficacy gains, although clinical translation requires GSH gradient validation (143) (Figure 3).

## 5 Current evidence: preclinical and clinical studies

### 5.1 Curcumin SLNs

A pilot clinical investigation examined the effects of 10 nm curcumin nanomicelles administered at a dose of 80 mg on alternate days for 2 months on oxidative stress–related pathways and serum cholinesterase levels in patients with AD compared with healthy individuals. The study reported no statistically significant changes in cognitive performance, as assessed by MMSE, or alterations in antioxidant enzyme activity or cholinesterase levels either before or after intervention. Notably, treatment with curcumin nanomicelles did not induce lipid peroxidation, indicating an acceptable safety profile (144). Savall et al. assessed the therapeutic efficacy of NC Curc in addressing cognitive deficits, oxidative damage, and neuroinflammation in an STZ-induced AD rat model. Oral administration of NC Curc markedly enhanced memory performance, suppressed acetylcholinesterase activity, alleviated oxidative stress, and restored GFAP expression to near-normal levels, reflecting the attenuation of neuroinflammatory responses. These outcomes were significantly superior to those observed with non-encapsulated curcumin, underscoring the enhanced neurorestorative potential of nanoformulated curcumin for AD management (145). Similarly, Sadegh Malvajerd et al. explored Cur-NLCs as a strategy to improve the cerebral delivery and therapeutic efficacy of curcumin in an Aβ-induced AD rat model. Both prophylactic and therapeutic administration of Cur-NLCs effectively counteracted Aβ-mediated cognitive dysfunction and neuronal toxicity, demonstrating their robust neuroprotective activity (146). Furthermore, Prathipati et al. investigated the protective effects of Cur-SLNPs against HCY-induced oxidative injury in a VaD rat model. Treatment with a higher dose of Cur-SLNPs (25 mg/kg) resulted in significant improvements in behavioral outcomes, biochemical indices, neurochemical parameters, and histopathological features associated with oxidative stress in multiple brain regions, highlighting the potential of SLNP-based curcumin delivery in VaD (147).

### 5.2 Resveratrol PLGA nanoparticles

A comparative investigation evaluated the neuroprotective efficacy of resveratrol-loaded nanoparticles administered via the oral and intranasal routes in an EAE mouse model. Treatment with RN delivered orally at 16.9 mg/kg or intranasally at 8.44 mg/kg resulted in a significant attenuation of RGC degeneration relative to untreated controls, with both administration routes exhibiting comparable protective outcomes. However, only oral RN reduced the severity of ascending paralysis and loss of visual function and improved survival outcomes. Neither administration route significantly reduced spinal cord or optic nerve inflammation, or demyelination. Overall, RN provides neuroprotection against RGC loss via both routes, with oral delivery offering additional benefits in reducing disease severity (148). Furthermore, J.-T. Yang et al. engineered phospholipid–polymer nanoparticles (PNPs) co-encapsulating RES and CEF with surface modification using leptin and transferrin to enable targeted brain delivery for the management of PD. The optimized nanoparticles improved drug brain permeation, protected dopaminergic cells from toxin-induced damage, and modulated key signaling pathways by upregulating neuroprotective proteins and downregulating neurodegenerative markers, such as *α*-synuclein and phosphorylated tau (149). Moreover, Marino et al. designed an eco-friendly antioxidant nanoplatform based on polyphenol-enriched grape pomace extracts encapsulated within surface-functionalized liposomes that exhibited enhanced BBB permeability. In an *in vitro* PD model, this nanoplatform markedly attenuated oxidative stress, inhibited α-syn aggregation, and restored cellular viability, underscoring its potential as a neuroprotective strategy (150). Consistently, low-dose intranasal administration of RNs has been investigated for neuroprotection in an EAE mouse model of MS. Intranasal RNs significantly improved RGC survival at approximately half the oral dose, thereby minimizing systemic exposure. Notably, despite persistent optic nerve inflammation and demyelination, RN treatment effectively preserves RGC integrity (151).

### 5.3 Quercetin nanoemulsion

Oliveira et al. evaluated PCL-based nanoparticles co-encapsulating quercetin and biapigenin to enhance neuroprotection against oxidative damage and improve brain delivery. The formulated nanoparticles significantly attenuated t-BOOH–induced cytotoxicity across multiple brain-derived cell lines and exhibited superior permeability across an *in vitro* BBB model (Papp ≈ 80 × 10^−6^ cm/s) relative to unencapsulated phytoconstituents (Papp ≈ 50 × 10^−6^ cm/s) (152). Chen *et al.,* developed glucose-modified quercetin liposomes (QU-Glu-Lip) to improve the poor solubility, bioavailability, and brain delivery of quercetin. The liposomes showed a high encapsulation efficiency (~90%), stable size (~120 nm), and good cytocompatibility. QU-Glu-Lip crosses the blood–brain barrier via GLUT1 transporters and is better taken up by neuronal cells than are non-glucose liposomes. They effectively reduce oxidative damage in neuronal cells, increase cell viability, and decrease reactive oxygen species (103). Sonawane and Pokharkar reported the development of quercetin-loaded NLCs (QT-NLC) integrated into an intranasal *in situ* gel to enhance cerebral delivery and therapeutic efficacy against neurodegeneration in rats. The optimized system exhibited a high encapsulation efficiency (~96%), nanoscale particle size (~123 nm), and sustained-release profile. Pharmacokinetic evaluation revealed markedly improved brain targeting, characterized by elevated intracerebral drug levels, and enhanced targeting efficiency. In an experimental neurodegeneration model, the QT-NLC gel produced significant cognitive improvement and demonstrated superior anti-AD efficacy compared to donepezil, underscoring its promise as an effective strategy for AD management (153) (Table 3).

**Table 3:** Nanocarrier approaches for neuroprotection.

Phytochemical	Nanocarrier	Disease model	Outcome	References
*Curcumin*	Nanomicelles (~10 nm)	Clinical trial - Alzheimer’s disease (patients, 80 mg, alternate days × 2 months)	No significant improvement in MMSE or antioxidant enzymes; no lipid peroxidation; safe profile but highlights need for optimized nanocarrier (larger particle size, improved concentration, BBB targeting)	(144)
*Curcumin*	Nanoencapsulated *curcumin* (NC Curc)	Streptozotocin (STZ)-induced Alzheimer’s disease rat model	Oral NC Curc significantly improved memory, reduced acetylcholinesterase activity, mitigated oxidative stress, and normalized GFAP levels, indicating reduced neuroinflammation; superior to unencapsulated *curcumin*.	(145)
Resveratrol (co-delivered with Ceftriaxone)	Phospholipid-polymer nanoparticles (PNPs), surface-functionalized with leptin & transferrin	Parkinson’s disease (cell and rat models)	Enhanced brain permeation; protected dopaminergic neurons; ↓α-synuclein & phosphorylated tau; ↑neuroprotective proteins; reduced apoptosis & neurodegeneration	(149)
Polyphenol-rich grape pomace extract	Functionalized liposomes (antioxidant nanoplatform)	Parkinson’s disease (in vitro model)	Enhanced BBB penetration; reduced oxidative stress; prevented α-synuclein aggregation; restored cell viability	(150)
Quercetin	Free compound (non-encapsulated)	Global cerebral ischemia–reperfusion (I/R) injury in rats	Reduced brain edema and BBB leakage; improved BBB integrity; ↑tight junction proteins (ZO-1, Claudin-5) and Wnt/β-catenin signaling; ↓MMP-9, GSK-3β, Axin; protective effects reversed by DKK-1 → confirming Wnt/β-catenin pathway involvement	(191)
Quercetin + Biapigenin	Poly(Ɛ-caprolactone) (PCL) nanoparticles	In vitro neurodegeneration models (brain cell lines & BBB model)	Reduced oxidative stress toxicity (t-BOOH); higher BBB permeability (Papp ~80 × 10^−6^ cm/s vs. ~ 50 × 10^−6^ for free drugs); demonstrated neuroprotective activity	(152)

### 5.4 Clinical evidence and translational status

Clinical evidence for nanoengineered phytochemical formulations is limited and concentrated in a small number of curcumin formulations tested in humans. Most efficacy claims in the literature derive from animal experiments and *in vitro* work; where human pharmacokinetic or interventional data exist we report them separately below and avoid extrapolating preclinical effect sizes to expected clinical outcomes. Systematic and comparative pharmacokinetic studies show that specially formulated curcumin products can increase plasma exposure relative to unformulated curcumin, but reported relative-bioavailability figures vary by formulation and study design (154, 155). Most efficacy signals summarized in this review arise from preclinical models; human data are concentrated in a limited set of curcumin formulations and remain heterogeneous in design and outcome measures. Systematic reviews and comparative PK studies confirm that specialized curcumin preparations can increase systemic exposure versus unformulated curcumin, but the magnitude of the increase depends strongly on formulation type, dose, and the analytic method used to quantify free versus conjugated curcuminoids. Importantly, few randomized, placebo-controlled trials have assessed cognitive or disease-relevant clinical endpoints for nanoengineered phytochemical products, and existing trials are small or exploratory. Consequently, claims about clinical benefit should be made cautiously and framed as hypothesis-generating rather than definitive (154, 156).

Table 4 lists completed human studies of nano- or bio-engineered phytochemical formulations that were identified in the search and reported in this manuscript. This table focuses on human PK, safety, and interventional outcomes so readers can distinguish preclinical signals from human evidence.

**Table 4:** Human PK and clinical studies of nanoengineered phytochemicals.

Compound (formulation)	Study type	*N* (per arm or total)	Dose and route	Primary endpoint(s)	Main outcome	References
Curcumin (Theracurmin®)	Randomized, double-blind RCT	40 (Theracurmin *n* = 21, placebo *n* = 19)	90 mg curcumin, oral, twice daily, 18 months	Memory and attention tests, FDDNP-PET amyloid/tau binding	Improved memory and attention vs. placebo; reduced FDDNP signal in some brain regions; small sample, exploratory PET subgroup.	(192)
Curcumin (Theracurmin®, phase I)	Phase I safety/PK in cancer patients	8–17 (dose cohorts)	Repeated oral dosing, escalating doses	Safety, PK (Cmax, AUC)	Acceptable safety in dose cohorts; improved systemic exposure relative to unformulated curcumin in PK analyses.	(193)
Curcumin (Longvida® SLCP)	Randomized, double-blind RCT	60–100 (varies by trial), examples *n* ≈ 60	400 mg/day Longvida®, oral, 4–12 weeks	Working memory, mood, cognition	Acute and short-term improvements in working memory and mood measures in healthy older adults, replicated in partial studies; clinical heterogeneity and small sample sizes.	(194)
Curcumin formulations (comparative PK)	Randomized crossover PK	small cohorts (*n* = 9–12)	Single dose, oral, crossover	Plasma curcuminoid AUC, Cmax	Comparative PK studies show large variability by product: some preparations (for example, Theracurmin, certain micellar/colloidal systems) report >10-fold increases in Cmax/AUC vs. standard curcumin in some studies; other products show more modest gains. Differences reflect dose, assay for free vs. total curcuminoids, and sample handling.	(195)
Curcumin (BCM-95 / Biocurcumax)	Pilot cross-over PK	12 (pilot)	Single dose, oral, crossover	AUC, Cmax	Early pilot data reported ~6–7 fold higher AUC vs. unformulated curcumin in one report; later comparative studies show heterogeneous results between studies and methods.	(196)
Resveratrol	Human nano-formulation trials: scarce	Mostly none or early PK pilots	Various	PK, safety	Human data for nanoformulations of phytochemicals other than curcumin are sparse. Most published translational studies for these compounds remain preclinical or limited to conventional (non-nano) formulations.	(197)

## 6 Challenges and limitations

### 6.1 Safety and toxicity of nanoparticles

Although nanocarriers represent highly effective platforms for brain-directed delivery and facilitate BBB traversal, several critical challenges remain. Nanosystems with extremely small dimensions may undergo unintended biodistribution, promote thrombus formation, or induce hemolysis, thereby triggering platelet aggregation. Moreover, non-uniform accumulation of NPs within distinct brain regions may pose substantial safety concerns. The presence of inorganic components in certain nanostructures, including Au-, silica-, Fe, and CeO₂-based particles, further complicates their metabolic fate and clearance. Persistent deposition of such materials in neural tissues may elicit neurotoxic outcomes by disrupting mitochondrial function and interfering with autophagy, apoptotic pathways, and neuroinflammatory signaling (157). Notably, NPs with a diameter of approximately 100 nm have been associated with potential CNS toxicity (158). Beyond their deliberate use in treating brain disorders, nanomaterials may also access the CNS unintentionally, either via systemic circulation following peripheral therapeutic applications or through inhalation exposure from the environment. Despite remarkable advances in nanomedicine, concerns regarding nanomaterial-induced neurotoxicity have emerged as a significant safety issue. The predominant mechanisms underlying NP-associated neurotoxicity include excessive oxidative stress, activation of apoptosis and autophagy, and stimulation of immune and inflammatory responses, all of which can collectively perturb BBB integrity and function (159).

It is important to recognize that oxidative stress in the CNS is not solely pathological. Reactive oxygen species (ROS) function as tightly regulated signaling mediators that modulate synaptic plasticity, mitochondrial dynamics, neuroinflammatory responses, and transcriptional programs such as Nrf2/Keap1-dependent antioxidant defense. Physiological redox signaling operates within defined spatial and temporal limits, whereas sustained ROS overproduction overwhelms antioxidant buffering systems and causes lipid peroxidation, protein carbonylation, DNA damage, and neuronal dysfunction. Consequently, the therapeutic objective of antioxidant phytochemicals is not indiscriminate ROS elimination but restoration of redox equilibrium (160, 161). Nanoformulations can influence this balance by modifying intracellular concentration gradients, subcellular localization, and release kinetics. While enhanced brain delivery may strengthen adaptive redox signaling pathways, excessive local accumulation or nanoparticle-induced surface reactivity may paradoxically intensify oxidative injury (162, 163). Therefore, mechanistic evaluation of nano-phytomedicines should differentiate between attenuation of oxidative damage and modulation of redox-dependent signaling cascades, particularly those governing neuronal survival and mitochondrial homeostasis.

Additionally, neurotoxic effects may directly compromise neuronal architecture or activity or indirectly propagate through glial activation and altered glia–neuron interactions. Such effects may present acutely or following prolonged exposure, with outcomes ranging from reversible dysfunction to permanent damage affecting localized brain regions or the CNS as a whole (164). Long-term safety requires explicit attention because several carrier classes show persistent brain retention and immune engagement that can evolve into chronic pathology. PAMAM and related dendrimers accumulate in injured brain regions and concentrate within activated microglia in many animal models, a property exploited for targeted therapy but one that also raises the possibility of cumulative dosing effects and sustained microglial stimulation (165, 166). Surface charge and terminal groups strongly influence this biology. Cationic surfaces and high-generation amine-terminated dendrimers increase membrane disruption, complement activation, and pro-inflammatory cytokine production *in vitro* and *in vivo*, whereas neutral or anionic surface chemistries show substantially lower acute toxicity (167, 168).

Persistent or repeated exposure to non-biodegradable or slowly cleared nanomaterials may therefore shift an initially adaptive microglial response toward chronic neuroinflammation, synaptic dysfunction, and neurodegeneration, particularly in aged or disease-susceptible brains (169). Current preclinical literature is divergent because many studies report short follow-up intervals and lack systematic measurement of long-term endpoints such as monthly biodistribution, microglial phenotyping (for example Iba1, CD68, MHC-II), cytokine panels, neuronal loss, and behavior. For translational confidence, studies should include prolonged follow-up (weeks to months), dose-fractionation schedules that mimic chronic dosing, aged animals or disease models, and paired clearance experiments to quantify retention and excretion routes (170). Practical mitigation strategies are available and should be emphasized in the discussion. These include using biodegradable backbones or enzymatically cleavable linkers, surface neutralization (for example hydroxylation, carboxylation or PEGylation) to reduce cationic surface reactivity, minimizing generation/size for dendrimers while preserving targeting, and requiring chronic-dosing safety packages that measure persistent microglial activation and behavioral outcomes before first-in-human trials. Studies that pair therapeutic efficacy with quantitative, long-term safety readouts will be decisive for clinical translation (167, 171).

Schneider conducted research which revealed that titanium dioxide (TiO2) and carbon black (CB) nanoparticles can bind to the neuronal cellular prion protein (PrP^C), disrupting its signaling and leading to overproduction of reactive oxygen species via NADPH oxidase. Oxidative stress and signaling disruption increase neuronal vulnerability to inflammation and promote excess neurotoxic amyloid-*β* peptides, which are key in the pathology of Alzheimer’s disease (AD) pathology (172). Furthermore, Larner et al. assessed the neurotoxic potential of several engineered nanoparticles, including SWNTs, C60 fullerenes, CdSe quantum dots, carbon black (CB), and dye-doped silica nanospheres (NSs), using PC-12 neuronal cell models. The results showed cytotoxicity at specific concentrations, with higher doses of SWNTs, CB, and C60 causing cell death, membrane damage, and vacuole formation. Differentiated PC-12 cells are more sensitive to nanoparticle toxicity, especially to CB and CdSe (173). Sharma et al. investigated the neurotoxic effects of metal-based NPs, including aluminum, silver, and copper, administered via multiple routes in rats and mice. This study demonstrated that these NPs compromised BBB integrity, decreased cerebral blood flow, and induced brain edema, neuronal damage, glial activation, upregulation of heat shock proteins, and myelin degeneration. Among the tested metals, Cu and Ag NPs produced the most pronounced neurotoxicity, particularly in murine models (174). These findings highlight the potential neurotoxic effects of these nanoparticles, particularly in neuron-like cells.

### 6.2 Standardization and quality control challenges

The standardization of herbal drugs refers to the process of adjusting herbal preparations to contain a defined amount of bioactive constituents with established therapeutic activity. This ensured consistent quality, precise dosage, and predictable therapeutic efficacy for each administered unit. The therapeutic effectiveness of herbal medicines is typically attributed to the synergistic interactions between multiple bioactive compounds, highlighting the importance of their standardization (175). In the context of nanomaterials, factors such as particle size, dosage regimen, administration route, surface chemistry, degree of aggregation, transmembrane diffusivity, excretion mechanisms, and immunogenic potential are the key determinants of nanotoxicity. Careful control of these parameters can modulate the interactions between nanomaterials and biological tissues, including their penetration, diffusion, absorption, distribution, immune recognition, retention time in organs, and systemic clearance, thereby minimizing unintended toxic effects (176).

### 6.3 Regulatory classification and approval barriers

India has a longstanding heritage of herbal medicine, with regulatory oversight of herbal products provided under the Drugs and Cosmetics Act (1940) and the AYUSH Ministry, which encompasses Ayurveda, Yoga and Naturopathy, Unani, Siddha, and Homeopathy. However, many traditional products are exempt from extensive clinical trials if they are classified as traditional or Ayurvedic. Herbal drug manufacturers must comply with GMP standards and the Ministry of AYUSH provides specific guidelines for the manufacture of herbal medicines. As in the EU, herbal products that are used for long-standing practices may be registered based on traditional knowledge and use without requiring modern clinical evidence (177). The US FDA serves as a critical regulator for healthcare products, including nanomedicines, by evaluating their safety and efficacy. Nanoscale materials possess distinct risk profiles owing to their small size, enhanced biological reactivity, and unique physicochemical properties. These largely uncharacterised risks introduce novel legal, ethical, and regulatory challenges for clinical trials, patient administration, and public health management (178). Although herbal medicines are often assumed to be inherently safe owing to their natural origin, several pose potential adverse effects, either directly or through herb–drug interactions with co-administered pharmaceuticals. Contributing factors include inaccurate labeling, unknown or variable composition, lack of standardization, substandard quality, contamination, adulteration, inappropriate use, and fraudulent practices. Consequently, integrating the pharmacovigilance of herbal medicines with that of conventional drugs within national pharmacovigilance frameworks is essential for ensuring patient safety and effective monitoring (179).

### 6.4 Scale-up and GMP constraints

Herbal drug manufacturers must comply with GMP standards, and regulatory bodies such as the Ministry of AYUSH provide guidelines to ensure quality and safety in herbal medicine production (177). GMP implementation minimizes batch-to-batch variability, reduces production errors, and enhances product credibility and regulatory acceptance (82). However, the issue of scaling up from laboratory development to industrial production may pose challenges in terms of reproducibility of particle size distribution, drug encapsulation efficiency, and stability, among others. Addressing these manufacturing constraints is essential for the successful translation of nano-enabled herbal therapeutics into clinically viable products. Although preclinical models demonstrate promising efficacy such as bacoside-loaded PLGA nanoparticles reversing scopolamine-induced cognitive deficits clinical evidence remains limited, with Phase I/II studies reporting modest therapeutic gains (92–94). To enhance clinical outcomes while ensuring product standardization and safety, these findings suggests the need for GMP-driven manufacturing optimization and rational combination strategies.

## 7 Future perspectives

### 7.1 Smart nanocarriers

Stimuli-responsive nanocarriers capitalize on the unique biochemical signatures of neurological disorders. For instance, pH-sensitive systems exploit the acidic milieu of inflamed or ischemic brain regions (pH 6.5–6.8) and endolysosomal compartments (pH 4.5–6.0) to trigger disassembly of polymeric micelles, liposomes, or dendrimer hybrids loaded with curcumin, resveratrol, or withanolides. Histidine-rich polymers or hydrazone linkages that protonate and swell at low pH ensure burst release precisely where oxidative stress and protein aggregation demand antioxidant intervention, minimizing systemic exposure and off-target effects (139). Similarly, redox-responsive carriers incorporating disulfide bridges (cleaved by elevated glutathione in activated microglia or neurons) liberate bacosides or quercetin exactly at sites of neuroinflammation, amplifying Nrf2 activation and neuroprotection while remaining inert during circulation (180).

Theranostic nanomedicine is emerging as a pivotal approach to personalized therapy. The integration of nanotechnology into theranostics has enabled the development of multifunctional nanoplatforms capable of both diagnostic imaging and targeted drug delivery, thereby enhancing detection and treatment of neurodegenerative disorders. Utilizing nanoparticles improves drug bioavailability and therapeutic efficacy, while minimizing systemic toxicity and adverse effects. Moreover, these nanosystems have demonstrated enhanced overall performance in both *in vitro* and *in vivo* evaluations, underscoring their potential for optimizing treatment outcomes (181). Multimodal theranostics integrates these responsive elements with imaging modalities for closed-loop delivery. Gadolinium- or SPIO-doped PLGA nanoparticles surface-functionalized with Angiopep-2 and loaded with curcumin could enable MRI tracking of BBB crossing and plaque targeting, with NIR-triggered release via embedded gold nanorods providing on-demand dosing. Fluorescence-quenched dendrimer-phytochemical conjugates will “light up” upon successful endosomal escape, confirming cytosolic delivery via confocal imaging (182).

### 7.2 AI and machine learning for predictive nanocarrier design

Nanoparticle (NP) platforms are gaining significant attention as carriers for delivering therapeutics to the central nervous system in NDDs. However, the development of effective Nanoparticle Neuronal Disease Drug Delivery Systems (N2D3S) is complex because of the vast combinatorial space of NPs and NDDS formulations, coupled with diverse assay requirements. AI/ML algorithms offer a promising solution for experimental evaluations by predicting the most promising NP and NDDS candidates. Nonetheless, the relatively limited datasets on N2D3S activity compared to the number of assayed NDD compounds poses challenges for AI/ML model training and predictive accuracy (183). Advanced programmed NP systems for theranostic delivery across the BBB are often conceptualized as “nanorobots” or “nanobots,” as they are engineered to perform specific functions with controlled navigation and targeted action (184). Nanobots are designed to traverse the BBB via multiple pathways, enabling the precise transport of drugs or nutrients into endothelial cells. Passive diffusion for CNS drug delivery is highly constrained because of the selective permeability of the BBB, which is maintained by tight junctions, adherens junctions, and the phospholipid bilayer of endothelial cells. Successful passive delivery is largely restricted to small lipophilic molecules, with efficiency strongly dependent on the molecular size, charge, surface area, and volumetric properties (185). For example, the IFPTML technique (Information Fusion-Perturbation Theory-Machine Learning) was applied to neuro drug delivery system (NDDS) data (4,403 assays from ChEMBL and 260 cytotoxicity assays). After resampling into 500,000-case datasets, linear (LDA) and nonlinear (MLP, DLN) models were developed. IFPTML-LDA achieved 70–73% sensitivity and specificity, while IFPTML-MLP/DLN reached 85–86% Sn/Sp with an AUROC 0.93–0.95, demonstrating strong predictive performance. These models highlight IFPTML’s potential of IFPTML as a computational tool for designing drug delivery systems in neuroscience (183).

### 7.3 Personalized nano-phytomedicine

Personalized nanomedicine represents a transformative approach in healthcare that integrates the precision of nanotechnology with patient-specific treatment strategies. This paradigm employs nanomaterials and nanostructures to develop targeted therapeutics tailored to the unique genetic, physiological, and biochemical profiles of individual patients (186). Moreover, nanomedicine can mitigate two major sources of variability in individualized drug responses: differences in cytochrome (CYP) enzyme activity and variations in drug transporter expression across populations. By enhancing the binding affinity, improving bioavailability, ensuring biocompatibility, and enabling controlled drug release, personalized nanomedicine maximizes therapeutic efficacy while delivering the drug to the intended target at the optimal time for each patient. Additionally, it facilitates integration with genomic insights, supporting the design of precise diagnostic and therapeutic strategies. Laboratory-engineered nanoparticles have already demonstrated significant promise, suggesting that the broader adoption of nanomedicine in personalized healthcare may be imminent. Collectively, nanomedicine and personalized medicine offer extensive potential to redefine the future landscape of therapeutics (187).

### 7.4 Integration with advanced techniques

Amid the growing demand for innovative therapeutic strategies, preclinical drug testing and screening platforms have undergone rapid advancement in recent years. Compared with conventional 2D cell cultures, 3D organoids or spheroids, either scaffold-based or scaffold-free, better recapitulate the *in vivo* microenvironment, enhance biological relevance, and enable mechanistic investigations of drug responses in a tissue-mimetic context. These 3D models are relatively straightforward to generate and exhibit consistent sizes and morphologies, facilitating reproducibility in experimental studies (188). 3D brain organoids derived from human pluripotent stem cells (hPSCs), including embryonic stem cells (ESCs) and induced pluripotent stem cells (iPSCs), replicate the cytoarchitectural complexity of the human brain and offer unprecedented opportunities to investigate disease pathogenesis. Patient-specific hiPSC-derived brain organoids serve as promising preclinical platforms to bridge the translational gap between animal models and clinical studies. Research employing patient-derived brain organoids has uncovered novel molecular and genetic mechanisms underlying complex neurological disorders, such as microcephaly, autism spectrum disorder, and Alzheimer’s disease. Integration of hiPSC technology with small-molecule high-throughput screening (HTS) facilitates the identification of novel pharmacotherapeutic agents, whereas transcriptomic profiling enables comprehensive gene expression analysis of patient-specific organoids. Moreover, combining these approaches with CRISPR/Cas9 genome editing offers transformative potential for personalized cell replacement therapies by enabling the precise genetic correction of hiPSCs, paving the way for individualized regenerative medicine strategies (189).

## 8 Conclusion

Neurodegenerative disorders continue to pose an immense clinical and societal challenge, largely because effective neuroprotective agents fail to reach the brain at sufficient concentrations to exert meaningful therapeutic effects. This review demonstrates that the limited success of many phytochemicals is not due to a lack of biological activity, but rather due to pharmacokinetic and delivery barriers, most notably poor bioavailability and restricted transport across the blood–brain barrier. Nanoengineered delivery systems provide a practical and scientifically grounded solution for these challenges by protecting labile phytochemicals, improving systemic exposure, and enabling targeted brain delivery. In multiple neurodegenerative disease models, lipid-based nanoparticles, polymeric systems, vesicular carriers, and dendritic platforms consistently enhance brain accumulation and neuroprotective efficacy when rationally designed. Key determinants of success include nanoscale size optimization, surface charge modulation, and ligand-mediated targeting, which exploit endogenous transport pathways at the blood–brain barrier. Importantly, the comparative evidence presented here indicates that nanocarrier performance is not uniform and that thoughtful engineering, rather than the mere use of nanotechnology, ultimately governs therapeutic outcomes.

Despite encouraging preclinical data, its translation into clinical practice remains limited. Safety concerns related to long-term nanoparticle exposure, variability in herbal extract composition, and the absence of harmonized regulatory frameworks for herbal nanomedicines represent significant barriers to clinical adoption. Addressing these challenges will require standardized phytochemical characterization, rigorous toxicity assessment, and a closer alignment between nanotechnology development and regulatory science. The integration of smart, stimuli-responsive nanocarriers with advanced screening tools, including artificial intelligence–guided formulation design and human-relevant brain models, offers a promising path forward. Such approaches may accelerate the identification of safe and effective brain-targeting phytochemicals. Ultimately, nano-phytomedicine should be viewed not as an alternative to conventional neurotherapeutics but as a complementary, design-driven strategy capable of translating the rich neuroprotective potential of plant-derived compounds into clinically meaningful interventions for neurodegenerative disorders.
